# Prognostic value of circulating vitamin D binding protein, total, free and bioavailable 25-hydroxy vitamin D in patients with colorectal cancer

**DOI:** 10.18632/oncotarget.16597

**Published:** 2017-03-27

**Authors:** Lin Yang, Hong Chen, Miao Zhao, Peng Peng

**Affiliations:** ^1^ Department of Gastroenterology, Zhongda Hospital, School of Medicine, Southeast University, Nanjing, Jiangsu, China

**Keywords:** VDBP, 25(OH)D, colorectal cancer, prognosis, overall survival

## Abstract

Numerous studies have suggested that there was a significantly positive association between circulating total 25-hydroxyvitamin D (25(OH)D) and survival in colorectal cancer patients. Moreover, plasma vitamin D was also found significantly associated with the concentration of vitamin D binding protein (VDBP). However, there was no paper to clarify the prognostic value of VDBP, free and bioavailable 25(OH)D in colorectal carcinogenesis. The aim of this study was to comprehensively assess the prognostic value of VDBP, total, free and bioavailable 25(OH)D in stage I-III colorectal cancer patients. A total of 206 colorectal cancer patients were enrolled in this prospective study. Preoperative plasma total 25(OH)D and VDBP concentrations were measured by direct enzyme-linked immunosorbent assay, and albumin concentration was measured by Beckman automatic biochemical analyzer. Free and bioavailable 25(OH)D concentrations were calculated based on the concentrations of plasma VDBP, total 25(OH) D and albumin. X-title program was used to determine the optimal cut-off values of VDBP, total, free and bioavailable 25(OH)D. Results showed that elevated free and bioavailable 25(OH)D were significantly associated with better 5-year overall survival (OS) by univariate analysis. By multivariate cox analysis, we also found that the high level of free 25(OH)D (HR = 0.442, 95%CI = 0.238–0.819, *P* < 0.010) could be identified as an independent factor for better OS. In conclusion, our study suggested that higher levels of free and bioavailable 25(OH)D were associated with better OS in stage I-III colorectal cancer patients. Moreover, free 25(OH)D could be considered as an independent prognostic biomarker for OS.

## INTRODUCTION

Colorectal cancer (CRC) is the third prevalent cancer and the fourth most common cause of cancer-related mortality worldwide [1]. In China, about 376.3 thousands people were diagnosed as CRC and about 191.0 thousands patients were died of CRC annually [2]. With the development of technology of diagnosis and treatment, many patients could be treated at the early stage of the disease, but the mortality of CRC was still increasing. Numerous studies have shown that high level of circulating vitamin D was associated with decreased CRC risk [3–7], suggesting a protective role of vitamin D against CRC. Although vitamin D can obtain from diet and even dietary supplements, most vitamin D is photochemically synthesized in the skin exposed to sunlight [8]. Unfortunately, due to changing of lifestyle and environment, reduced outdoor activity and the absence of adequate sun exposure, vitamin D deficiency becomes a common phenomenon both in healthy individuals and in CRC populations [9].

Vitamin D, a kind of trace elements, exists with two major kinds of formations, 25-hydroxy vitamin D (25(OH)D,) and 1,25-dihydroxy vitamin D_3_ (1,25(OH)_2_D_3_). Particularly, 25(OH)D, is the major circulating form of vitamin D[10], thus, the total serum 25(OH)D level is considered to be the best indicator of vitamin D [11]. Transported from liver to circulation, 25(OH)D is bound primarily to vitamin D binding protein (VDBP) and albumin, and a very small fraction remains free or unbound [12]. The latter is called as free 25(OH)D. Besides, albumin shows a weaker affinity to 25(OH)D than that of VDBP, thus, 25(OH)D can easily dissociate from the albumin [13]. Therefore, both free and albumin bound 25(OH)D can bind to vitamin D receptor (VDR) easily in the surface of target tissues and exert biological effects. As a result, free and albumin bound 25(OH)D are regarded as the bioavailable 25(OH)D. On the other hand, VDR is expressed both in normal cells and in malignant dividing cells[14], and is related to cell proliferation, differentiation, invasion and angiogenesis [15]. Moreover, VDBP is a multifunctional protein that belongs to the albumin gene family and is the precursor to the immune-modulatory protein[16], Gc protein-derived macrophage activating factor (Gc-MAF), which is a Macrophage Activating Factor (MAF) that can activate macrophage against cancer cell [17]. Hence, we speculated that the concentrations of peripheral blood vitamin D and VDBP were associated with the survival in patients with CRC.

Recently, several studies reported that 25(OH)D was associated with the risk of CRC [18–20], and VDBP was not associated with the risk of various cancers, including CRC [21]. However, VDBP was found significantly associated with the concentration of plasma vitamin D [22, 23]. Recently, Ying et al. demonstrated that there was no direct association between VDBP and the risk of CRC. And they also found low level of 25(OH)D was associated with the risk of CRC only when circulating VDBP was below the median level [24]. These results indicated that it might be better to combine 25(OH)D and VDBP together for describing the relationship between vitamin D and CRC. Furthermore, two studies have shown an association between low level of total 25(OH)D and poor CRC survival outcome [3, 8]. Unfortunately, all of these studies didn't further explore the prognostic value of free, bioavailable 25(OH)D and VDBP in CRC patients. Hence, in this study, we aimed to comprehensively assess the prognostic value of VDBP, total, free and bioavailable 25(OH)D in stage I-III CRC patients.

## RESULTS

### The optimal cut-off values of plasma VDBP, total, free and bioavailable 25(OH)D

**Figure 1:**
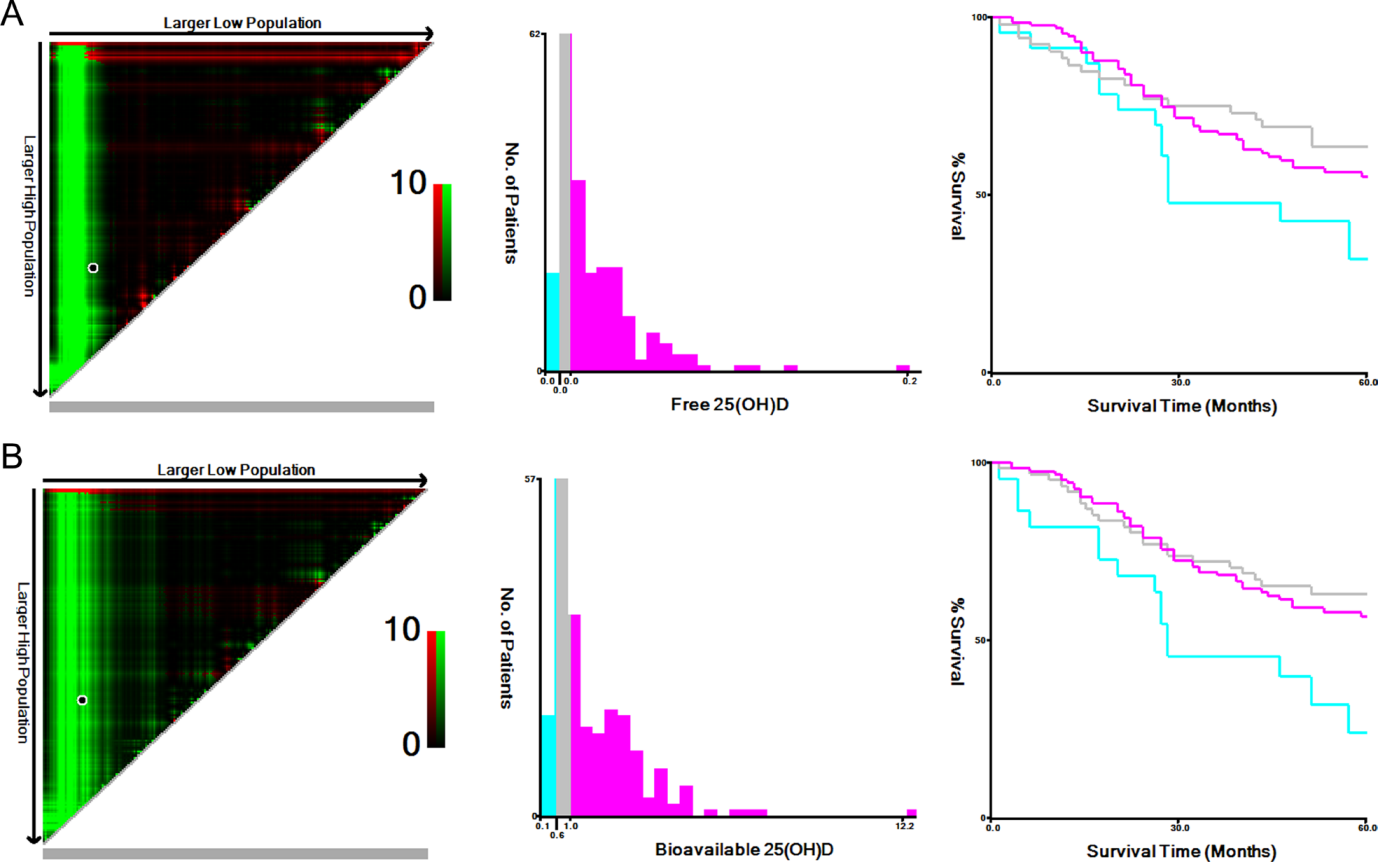
X-tile analyses for 5-year overall survival The sample of CRC patients was equally divided into training and validation sets. X-tile plots of training sets were shown in the left panels, with plots of matched validation sets shown in the smaller inset. The optimal cut-off values highlighted by the black circles in left rectangular panels were also shown in histograms of the entire cohort (middle panels), and Kaplan–Meier plots were displayed in right panels. *p* values were determined by using the cut-off values defined in training sets and applying them to validation sets. The optimal cut-off values of Free 25(OH)D and Bioavailable 25(OH)D for OS were 0.01–0.01 pg/ml, and 0.58–1.03 ng/ml, respectively. (**A**) Free 25(OH)D. (**B**) Bioavailable 25(OH)D. CRC, colorectal cancer; VDBP, vitamin D binding protein; OS, overall survival.

The patients were categorized into three tertiles based on the VDBP, total, free and bioavailable 25(OH)D determined by X-title program (Figure 1). The VDBP tertiles obtained from our data include the low VDBP tertile group (VDBP<159.2 ug/ml), middle VDBP tertile (VDBP = 159.2–310.1 ug/ml), the high VDBP tertile (VDBP > 310.1ug/ml). Likewise, total, free and bioavailable 25(OH)D tertiles also included low, middle and high tertile groups, which were listed in Table 1.

**Table 1:** The cut-off values of plasma VDBP, total, free and bioavailable 25(OH)D

Group	Low group	Middle group	High group
VDBP (ug/ml)	< 159.2	159.2–310.1	> 310.1
Total 25(OH)D (ng/ml)	< 6.2	6.2–29.9	> 29.9
Free 25(OH)D (pg/ml)	< 0.01	0.01–0.02	> 0.02
Bioavailable 25(OH)D (ng/ml)	< 0.58	0.58–1.03	> 1.03

Abbreviations: 25(OH)D: 25-hydroxyvitamin D; VDBP: vitamin D binding protein.

### Baseline characteristics of enrolled participants

The baseline characteristics of CRC patients were summarized in Table 2. A total of 206 CRC patients with 131 males and 75 females were enrolled in this study. They had a median age of 63 years (range from 30 to 85). Of them, no smoking, no drinking, no diabetes and no hypertension were all in the majority. Colon was the most common location site of the tumor (*n* = 112). A total of 125(60.7%) patients were in stage III, and according to local histopathology grading, 155(75.2%) patients were found in moderate cell differentiation. All patients underwent surgery and 151(73.3%) patients received chemotherapy. During follow-up period, 87 patients (42.2%) died by the date of the recently scheduled follow-up, the median follow-up time was 45 months.

**Table 2:** The baseline characteristics of patients with colorectal cancer

		VDBP				Total 25(OH)D				Free 25(OH)D				Bioavailable 25(OH)D			
Total patients	Low	Middle	High	P*	Low	Middle	High	P*	Low	Middle	High	P*	Low	Middle	High	P*
Parameters	*N* = 206	*N* = 40	*N* = 145		*N* = 21	*N* = 59	*N* = 123		*N* = 24	*N* = 19	*N* = 72		115	*N* = 21	*N* = 61		*N* = 124
Age(years)					0.037				0.116				0.555				0.68
> 63	110	25	79	6		38	59	13		9	42	59		13	31	66	
< 63	96	15	66	15		21	64	11		10	30	56		8	30	58	
Gender					0.57				0.07				0.061				0.023
Male	131	28	91	12		44	75	12		14	52	65		14	47	70	
Female	75	12	54	9		15	48	12		5	20	50		7	14	54	
Smoking					0.402				0.055				0.247				0.324
Yes	49	8	38	3		15	33	1		4	22	23		6	18	25	
No	157	32	107	18		44	90	23		15	50	92		15	43	99	
Drinking					0.321				0.371				0.401				0.89
Yes	24	3	20	1		6	17	1		1	11	12		2	8	14	
No	182	37	125	20		53	106	23		18	61	103		19	53	110	
BMI(kg/m^2^)					0.01				0.183				0.215				0.508
< 22.16	103	28	63	12		34	55	14		13	33	57		12	27	64	
> 22.16	103	12	82	9		25	68	10		6	39	58		9	34	60	
Hypertension					0.228				0.946				0.215				0.12
Yes	46	5	35	6		13	27	6		3	21	22		5	19	22	
No	160	35	110	15		46	96	18		16	51	93		16	42	102	
Diabetes					0.487				0.156				0.056				0
Yes	30	8	20	2		13	14	3		5	14	11		10	9	11	
No	176	32	125	19		46	109	21		14	58	104		11	52	113	
TMN stage					0.643				0.311				0.962				0.821
I-II	81	18	56	7		24	51	6		7	29	45		8	26	47	
III	125	22	89	14		35	72	18		12	43	70		13	35	77	
Cell differentiation					0.937				0.195				0.86				0.665
H	20	4	16	0		8	10	2		3	9	8		2	7	11	
M	155	30	106	19		39	101	15		13	49	93		14	44	97	
L	31	6	23	2		12	12	7		3	14	14		5	10	16	
Location					0.02				0.206				0.008				0.049
Colon	112	16	88	8		27	73	12		5	47	60		7	39	66	
Rectal	94	24	57	13		32	50	12		14	25	55		14	22	58	
Chem					0.205				0.605				0.818				0.06
Yes	151	33	105	13		42	93	16		13	52	86		11	48	92	
No	55	7	40	8		17	30	8		6	20	29		10	13	32	
Albumin (g/l)					0.003				0.097				0.136				0.025
< 35	69	11	44	14		15	42	12		6	18	45		12	15	42	
> 35	137	29	101	7		44	81	12		13	54	70		9	46	82	
Death					0.177				0.371				0.013				0.047
Yes	87	13	62	12		22	52	13		13	23	51		14	22	51	
No	119	27	83	9		37	71	11		6	49	64		7	39	73	

Abbreviations: 25(OH)D: 25-hydroxyvitamin D; VDBP: vitamin D binding protein; BMI: body mass index; Chem: Chemotherapy; H: high cell differentiation; M: moderate cell differentiation; L: low cell differentiation.

Note: The bold represent that *P* value was statistically significant.*Difference between groups was tested by Chi-square test.

### The association between baseline characteristics and clinical prognosis

The association between baseline characteristics and overall survival (OS) in CRC patients were listed in Table 3. In Kaplan–Meier and univariate analysis, our results showed that age (< 63 years), no smoking, body mass index (BMI) (< 22.16 kg/m^2^), no hypertension, no diabetes, TNM stage (I–II), high cell differentiation, albumin (> 35 g/l), middle and high group of free and bioavailable 25(OH)D were significantly associated with better OS(*P*_all_ < 0.05; Figure 1 and Table 3). Clinical baseline characteristics for the prediction of clinical prognosis were further investigated by multivariate analysis with Cox regression model. Smoking (HR = 1.841, 95%CI = 1.149–2.949, *P* = 0.011), BMI (> 22.16 kg/m^2^) (HR = 2.004, 95%CI = 1.244–3.228, *P* = 0.004), low cell differentiation (HR = 3.912, 95%CI = 1.130–13.551, *P* = 0.031), albumin (> 35 g/l) (HR = 0.590, 95%CI = 0.383–0.909, *P* = 0.017) were still associated with clinical prognosis. More importantly, comparing patients in the high group and middle group versus the low group of free 25(OH)D, the adjusted hazard ratio (HR) was 0.442(95%CI = 0.238–0.819, *P* = 0.010) and 0.241(95%CI = 0.117–0.495, *P* < 0.001) for OS, respectively. Our results indicated that middle and high group of free 25(OH)D, no smoking, BMI (< 22.16 kg/m^2^), high cell differentiation, albumin (> 35 g/l) could be considered to be independent markers for better OS of CRC patients.

**Table 3:** COX regresion based on overall survival

Parameters	Total patients	5-year OS	Log-rank *P* value	univariate analysis		multivariate analysis	
	*N* = 206	NUM of death		HR(95%CI)	*P*-value	HR(95%CI)	*P*-value
**Age(years)**			**0.047**				
< 63	96	49		Reference		Reference	
> 63	110	38		**1.528(1.000,2.334)**	**0.05**	1.237(0.766,1.999)	0.385
**Gender**			0.921				
Male	131	56		Reference			
Female	75	31		0.978(0.631,1.517)	0.922		
**Smoking**			**0.03**				
No	157	59		Reference		Reference	
Yes	49	28		**1.631(1.040,2.558)**	**0.033**	**1.841(1.149,2.949)**	**0.011**
**Drinking**			0.555				
No	182	75		Reference			
Yes	24	12		1.199(0.652,2.206)	0.559		
**BMI(kg/m^2^)**			**0.008**				
< 22.16	103	34		Reference		Reference	
> 22.16	103	53		**1.778(1.155,2.736)**	**0.009**	**2.004(1.244,3.228)**	**0.004**
**Hypertension**			**0.011**				
No	160	60		Reference		Reference	
Yes	46	27		**1.787(1.134,2.816)**	**0.012**	1.544(0.959,2.486)	0.074
**Diabetes**			**0.005**				
No	176	68		Reference		Reference	
Yes	30	19		**2.035(1.222,3.388)**	**0.006**	1.403(0.763,2.581)	0.276
**TMN stage**			**0.038**				
I-II	81	26		Reference		Reference	
III	125	61		**1.613(1.018,2.556)**	**0.042**	1.147(0.900,1.463)	0.267
**Cell differentiation**			**0.03**				
H	20	3		Reference		Reference	
M	155	67		**3.584(1.127,11.397)**	**0.031**	2.281(0.699,7.442)	0.172
L	31	17		**4.662(1.365,15.914)**	**0.014**	**3.912(1.130,13.551)**	**0.031**
**Location**			0.569				
Rectal	94	41		Reference			
Colon	112	46		0.886(0.581,1.350)	0.572		
Chem			0.365				
No	55	20		Reference			
Yes	151	67		1.257(0.763,2.073)	0.37		
**Albumin (g/l)**			**0.001**				
< 35	69	40		Reference		Reference	
> 35	137	47		**0.490(0.321,0.747)**	**0.001**	**0.590(0.383,0.909)**	**0.017**
**VDBP**			0.203				
Low	40	13		Reference			
Middle	145	62		1.463(0.805,2.661)	0.212		
High	21	12		2.013(0.918,4.416)	0.081		
**Total 25(OH)D**			0.23				
Low	59	22		Reference			
Middle	123	52		1.180(0.717,1.943)	0.516		
High	24	13		1.794(0.903,3.563)	0.095		
**Free 25(OH)D**			**0.008**				
Low	19	13		Reference		Reference	
Middle	72	23		**0.353(0.178,0.699)**	**0.003**	**0.241(0.117,0.495)**	**0**
High	115	51		**0.535(0.290,0.985)**	**0.045**	**0.442(0.238,0.819)**	**0.01**
**Bioavailable 25(OH)D**			**0.019**				
Low	21	14		Reference		Reference	
Middle	61	22		**0.410(0.210,0.803)**	**0.009**	0.807(0.327,1.992)	0.642
High	124	51		**0.485(0.268,0.877)**	**0.017**	0.398(0.082,1.928)	0.253

Abbreviations: OS: overall survival; HR: hazard ratio; 95%CI: 95% confidential interval; Chem: Chemotherapy; 25(OH)D: 25-hydroxyvitamin D; VDBP: vitamin D binding protein; ALB: albumin;BMI: body mass index; L: Low group; M: Middle group; H: High group.

Note: The bold represent that *P* value was statistically significant.

## DISCUSSION

In this study, we found that low levels of free and bioavailable 25(OH)D were both associated with poor OS, and free 25(OH)D was an independent prognostic factor for OS. To the best of our knowledge, this might be the first research which explored the association of plasma VDBP, total, free and bioavailable 25(OH)D with the prognosis of patients with stage I-III CRC at the same time.

This study indicated that low levels of free and bioavailable 25(OH)D were both associated with poor OS, which indicated that free and bioavailable 25(OH)D had a protective effect from death for CRC patients and had utility as biomarkers for survival monitoring. However, previous studies reported that total 25(OH)D was also associated with the survival of CRC [3, 8], which was inconsistent with our study. Maybe this phenomenon could be explained by the number of patients from different tumor stages and different geographic regions. Therefore, further study with larger samples should be applied to confirm this result. Furthermore, different cut-off values determined by different tools could also contribute to this difference. In this study, X-tile program, a robust graphical tool verified by Yale University was used by us to determine the optimum cut-off values. Our study demonstrated that circulating VDBP was not associated with the survival of CRC patients, which was consistent with the recent study by Ying et al. [24]. Theoretically, free and bioavailable 25(OH)D would be more representative for the function of vitamin D than total 25(OH)D because only free 25(OH)D could bind to VDR and play an important role in biological activity [25].

The following mechanisms might explain our results. Almost all CRC patients had abnormal β-catenin signal transduction pathway[26] and 25(OH)D in colon and several other tissues could be converted into hormonally active 1,25(OH)_2_D_3_ which could inhibit the abnormal signal pathway, and promote the differentiation of tumor cells by stimulating β-catenin-TCF-4 target gene [27, 28] and inducing E-cadherin [29, 30]. Furthermore, the 1,25(OH)_2_D_3_ had direct anti-tumor effect [31] because several oncogenes or anti-oncogenes could be regulated by vitamin D [32], such as TGF-β, TNF-α, p27/Kip1, c-myc, c-fos, c-jun, which were closely associated with the proliferation and differentiation of some specific cells [33]. Some previous studies indicated that TGF-β, which could inhibit the growth of enterocytes, could be antagonized by CRC cells[34], and this resulted in the growth of CRC tissues. However, this process could be interdicted by 1,25(OH)_2_D_3_ through increasing the activity of TGF-β on CRC cells[35]. In addition, 1,25(OH)_2_D_3_ could suppress mitosis by increasing the expression levels of p27/Kip1 and p21/Waf1, inhibiting the activity of cyclin-CDK complex, promoting dephosphorylation of Rb protein, decreasing the transactivation of E2F, and then resulting in the tumor cells stagnated in G0/G1 stage [36].

To our knowledge, it was the first study that synthetically investigated the association between total, free, bioavailable 25(OH)D, VDBP and the prognosis of patients with stage I-III CRC. The previous studies tended to be more concerned about the total 25(OH)D or VDBP with CRC outcome separately. Our study could objectively and comprehensively analysis the association among them and our results were then more accurate than the previous. Besides, all samples were collected between 8:00am and 10:00am in ZhongDa hospital, and the plasma samples were all put in -80 degree centigrade environment before collection for assays, which could minimize the side effects of environmental factors on plasma vitamin D levels.

Undeniably, some limitations were also found in our study. Firstly, only 206 patients were enrolled and the small size couldn't represent the whole population which might decrease the power of our results. So further study with more patients included should be conducted. Secondly, the concentrations of 25(OH)D and VDBP were assayed at only one point instead of dynamic monitoring, which might have an important side effect on our results because the plasma 25(OH)D level would increase by extral multivitamin use, physical activity, and exposure to sunshine. Thirdly, only OS was accessed in this study, which was not able to exclude the influence of cancer unrelated to death. The last but not least, there was a lack of an external validation cohort to test whether actively supplementing vitamin D could increase survival time of CRC patients.

In conclusion, our findings suggested that higher levels of free and bioavailable 25(OH)D were associated with better OS in I-III stage CRC patients, and free 25(OH)D was an independent prognostic factor for OS.

## MATERIALS AND METHODS

### Study population

A blanket search was conducted to look for the patients initially diagnosed with CRC from February 2011 to February 2012 in ZhongDa Hospital (Nanjing, Jiangsu, China). All patients were diagnosed through colonoscopy, confirmed with histo-pathological examination and underwent surgery. Patients with any diseases which could have an impact on the metabolism of vitamin D or calcium were excluded from our study, such as serious renal or liver diseases, parathyroid gland diseases, active infection, etc. The present study was approved by the Institution Ethics Commission of ZhongDa Hospital and Southeast University, and informed consents were obtained from all participants.

### Information of patients

We collected the characteristics of all enrolled patients, including sex, age, smoking, drinking, BMI, diabetes, hypertension, treated with surgery or chemotherapy, and the treatment data. In addition, the location of cancer, pathological types, tumor stage were also collected. All information above were obtained from the hospital medical record of ZhongDa Hospital.

### Blood sample saving and plasma vitamin D assay

All samples were collected within seven days before operation from Clinical Laboratory of ZhongDa Hospital. To minimize the effect of ultraviolet or temperature on blood samples, the collecting time was controlled between 8:00am and 10:00am, and the plasma samples were put in -80 degree centigrade environment until assay. The measurement of blood total 25(OH)D was conducted using direct competitive enzyme-linked immunosorbent assay with the 25(OH)D immunoassay kit (HCB Ltd, Vancouver, Canada). The concentration of plasma VDBP was measured using human VDBP immunoassay kit (R&D Systems, Minneapolis, USA). Considering the coefficient of variations of inter- and intra-batch about the two kinds of kit, all plasma samples were distributed to triplicate randomly while measuring. The measurement of plasma albumin concentration was also carried out using a bromocresol green dye assay with Beckman automatic biochemical analyzer (AU680) (Beckman Coulter lnc, Tokyo, Japan). After all above steps, free and bioavailable 25(OH)D were calculated from the concentrations of total 25(OH)D, VDBP and plasma albumin according to the mathematical equations provided by Bhan et al[37]. All assays were conducted in April of 2014. The methods were carried out according with the approved guidelines.

### Follow up

Patients were followed up regularly dating up to December 10, 2016 or until death according to 7th edition of the TNM-UICC/AJCC classification for CRC (every 3–6 months for first 2 years, every 6 months for the third to fifth years). The OS was defined as the time from the date of diagnosis to last follow-up or death. Follow-up data for patients were computed from medical records, physical examinations, laboratory examinations or telephone follow-up. All works were conducted by two investigators and any disagreement was solved by discussion.

### Statistical analysis

IBM SPSS Statistical 20.0 (SPSS Inc. Chicago, IL, USA) and X-tile 3.6.1 (Yale University, New Haven, CT, USA) were used for statistical analysis. Differences between high- and low-level group patients were evaluated using the χ2 test or Mann-Whitney U test. For clinical practice, the continuous variables were changed to categorical variables, and the cutoff values of VDBP, total, free and bioavailable 25(OH)D were determined by X-tile 3.6.1 (Yale University, New Haven, CT, USA). OS curves were established according to the Kaplan–Meier method and the differences were analyzed by the log-rank test. To identify the independent factors, multivariate cox regression analyses were performed. Variables with a *P value* less than 0.10 in the univariate cox regression analysis were entered into the multivariate cox regression model using backward conditional method. Two-tailed *P* values less than 0.05 were considered to be statistically significant.

## Abbreviations

25(OH)D: 25-hydroxyvitamin D
VDBP: vitamin D binding protein
CRC: colorectal cancer
OS: overall survival
1,25(OH)_2_D_3:_: 1,25-dihydroxy vitamin D_3;_
VDR: vitamin D receptor
Gc-MAF: Gc protein-derived macrophage activating factor
BMI: body mass index
HR: hazard ratio
95%CI: 95% confidential interval
